# Thermo-responsive plasmonic systems: old materials with new applications

**DOI:** 10.1039/c9na00800d

**Published:** 2020-03-18

**Authors:** Tao Ding, Jeremy J. Baumberg

**Affiliations:** Key Laboratory of Artificial Micro- and Nano-structures of Ministry of Education of China, School of Physics and Technology, Wuhan University Wuhan 430072 China t.ding@whu.edu.cn; NanoPhotonics Centre, Cavendish Laboratory, University of Cambridge Cambridge CB3 0HE UK

## Abstract

Thermo-responsive plasmonic systems of gold and poly(*N*-isopropylacrylamide) have been actively studied for several decades but this system keeps reinventing itself, with new concepts and applications which seed new fields. In this minireview, we show the latest few years development and applications of this intriguing system. We start from the basic working principles of this puzzling system which shows different plasmon shifts for even slightly different chemistries. We then present its applications to colloidal actuation, plasmon/meta-film tuning, and bioimaging and sensing. Finally we briefly summarize and propose several promising applications of the ongoing effort in this field.

## Abstract 2

Thermo-sensitive plasmonic system made of Au and poly(*N*-isopropylacrylamide) are exploited for various applications from optical tuning and chemical sensing to microfluidics and nanoactuation.

## Graphical abstract

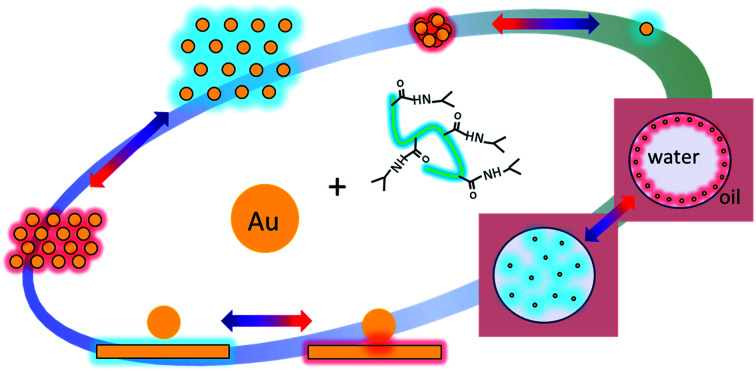

## 1. Introduction

Active plasmonics has attracted great interest in the plasmonics community and numerous efforts have been devoted to this with many emerging concepts and applications.^1,2^ Different from passive plasmonic nanostructures, which are solely made of metals with fixed configurations, active plasmonics combines metallic nanostructures with functional materials, which brings tuneability to the plasmonic system. With such active tuneability, active modulation of the light flow at the nanoscale is possible, which triggers a series of applications in information technologies,^3^ energy harvesting,^4^ (bio)chemical sensing^5,6^ and security.^7,8^ The functional materials used commonly include responsive polymers,^9^ DNA origami,^10^ liquid crystals^11^ and optoelectronic materials.^12^ This active plasmonics concept also links with nonlinear optics,^13^ ultrafast optics^14^ and quantum plasmonics^15^ to form many extended directions.

**Fig. 1:**
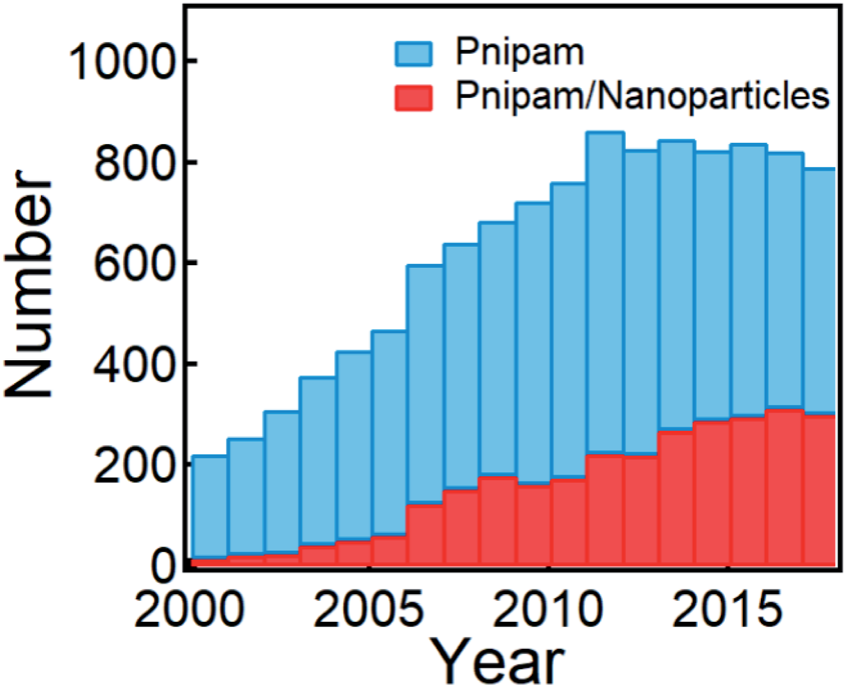
Publications that involve PNIPAM and PNIPAM/nanoparticles within the last two decades (from 2000 to 2018, search updated on December 12, 2019 from Web of Science).

Responsive (macro)molecules are typical materials that have been vigorously utilized in active plasmonics as they provide optical means for the (multiplexed) sensing of external stimuli, such as pH,^16,17^ temperature,^18,19^ light,^20,21^ electric^22,23^ and magnetic fields.^24^ One of the most studied stimulus materials are synthetic polymers showing a lower critical solution temperature (LCST) such as poly(*N*-isopropylacrylamide) (PNIPAM), which promotes reversible (de)solvation when the temperature is (above) below the LCST. This has been studied since the late 1990s, and is still actively investigated (Fig. 1) for different applications,^25^ such as sensing,^26^ catalysis,^27,28^ drug delivery,^29^ tissue engineering,^30^ artificial muscles,^31^*etc.*

Early research on PNIPAM mainly focused on block co-polymers containing PNIPAM segments or polymer core–shell hybrids.^32–34^ Later, this extended to inorganic cores such as silica,^35^ iron oxides^36^ and noble metals such as platinum for catalysis applications.^28^ With the development of nanophotonics in the 1990s, plasmonic nanoparticles (NPs) were also hybridized with PNIPAM.^37^ In this early research, the PNIPAM brushes/chains were either chemically grafted-from or grafted-to the metallic nanoparticle surfaces.^38–40^ On changing the temperature across the LCST of PNIPAM, the refractive index and volume of PNIPAM hydrogel changes, which modifies the optical plasmon resonances. Using this principle, many new designs and materials for thermo-responsive plasmonic systems bloomed.^41–43^ Although now well-known, this field is still developing and many new concepts and intriguing applications are still emerging,^44–50^ which deserve revisiting.

In this minireview, we introduce recent understanding and developments of such thermo-responsive plasmonic systems. Although silver (Ag) NPs may show better plasmonic performances,^51–53^ we mainly focus on the systems made of gold (Au) NPs and PNIPAM as the latter is more stable and reliable. Nevertheless, the working principle of thermal responsiveness is the same for both plasmonic NPs. We firstly analyse the physical chemistry aspects of this hybrid plasmonic system (Section 2), then recent applications for nanoactuation, plasmon tuning, metamaterials, and chemical sensing will be highlighted (Section 3). Lastly, we will conclude this minireview with a brief summary and perspectives of this growing field (Section 4).

## 2. Fundamental physical chemistry of Au NP/PNIPAM hybrid system

**Fig. 2:**
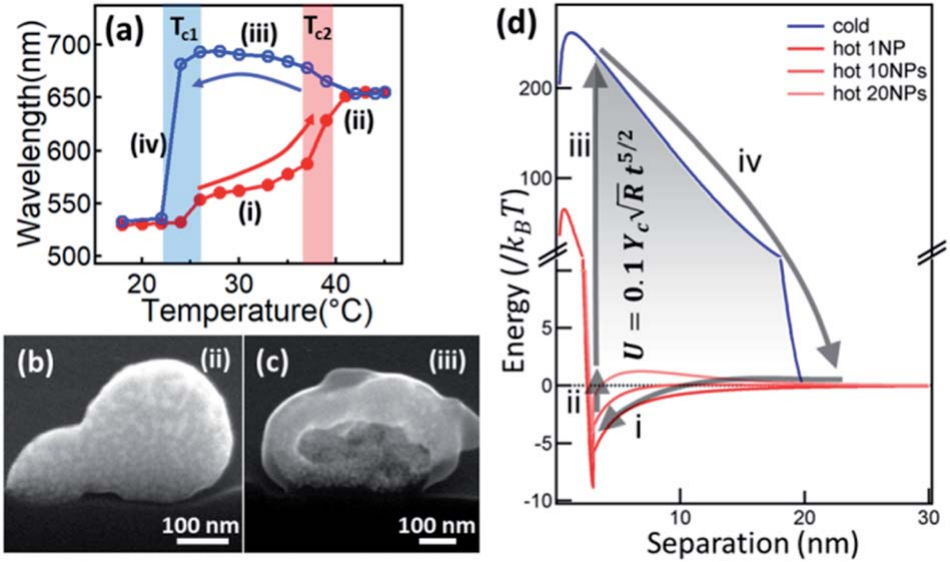
Physical understanding of the temperature-sensitive reversible assembly of Au@PNIPAM. (a) Plasmon resonance change with temperature (red on increasing *T*, blue on decreasing *T*). (b and c) SEM cross-sectional images of Au/PNIPAM clusters of different configurations at different stages (modified from ref. 60. Copyright permission from RSC). (d) Energy diagram of the Au/PNIPAM hybrid system with changing separation between the Au NPs. Red lines represents the aggregation of Au NPs while blue line represents the disaggregation. Process (i) happens when *T* > *T*_c_, and process (ii–iv) happen instantaneously when *T* decreases below *T*_c_. The energy stored in this cycle is expressed as the formula shown in the shaded region, where *γ*_c_ is Young's modulus of PNIAPM, *R* is radius of Au NPs, and *t* is thickness of PNIPAM coating (modified from ref. 45. Copyright permission from PNAS).

The temperature responsiveness of the Au/PNIPAM hybrid varies greatly in different systems. In most cases, only a very small plasmon shift^54–57^ or change in attenuation of transmission^58,59^ is observed when cycling the temperature while in other cases, the plasmon shift is very large.^45,60,61^ This inconsistency has recently been revealed as due to extra free PNIPAM in solution which is the main driving force promoting particle aggregation.^62,63^ Besides this, PNIPAM grafting density differences on Au surfaces also play some role, which has been thoroughly reviewed in previous literature.^64^ Beyond this, the electrostatic repulsion (*U*_cs_) between nanoparticles seems to be crucial as it limits the closest separation between the Au NPs.^65^ Particles are stabilized with high surface charges even though the PNIPAM chains coil around them.^60^ The hydration of the PNIPAM chains coated on the particles' surfaces provides another shielding even if the zeta potential (*Ψ*) is low. However, when the PNIPAM chains are triggered through the phase transition upon heating, the nanoparticle surface becomes hydrophobic, which leads to aggregation. This seemly simple aggregation is actually a variable and complicated process. In one sense this system is simply switching between dispersed Au NPs and aggregated Au NP clusters. However, if the pH is lower than 3, this Au/PNIPAM hybrid system shows two transitions in each heating and cooling cycle (Fig. 2a), which corresponds to the aggregation of Au NPs and co-aggregation of Au clusters and PNIPAM beads. Intriguingly, a redshift of plasmons is observed during the cooling cycle (stage iii), which is due to the transformation from solid clusters (Fig. 2b) to hollow vesicles (Fig. 2c).^60^ Clearly, the assembly pathways of AuNPs/PNIPAM hybrids can be altered by changing the aggregation kinetics of PNIPAM chains, which is related to the amount of PNIPAM, pH, and salt in this system.

The existence of a steric potential (*U*_e_) from the polymer chains makes the aggregation events reversible. This takes effect when the particle separation becomes smaller than the PNIPAM coating thickness, with steric hindrance working against the van de Waals potential (*U*_vdW_) to reach a new equilibrium.^66^ During cycles of this colloidal transition, significant amounts (∼1000 *k*_B_*T* per NP) of elastic energy in the polymer chains are stored and released, in a reversible energy cycle (Fig. 2d). The expansion force is thus large (∼nN) with fast dynamics of the polymer phase transition (∼μs).^45,67^ With such large forces and fast response, many novel applications emerge, such as plasmonic nanoactuators,^44–46,68,69^ plasmon tuning,^70,71^ fast plasmonic color switching films and metafilms,^49,72,73^ and plasmonic sensing.^16,47,48,74^ These will be discussed in the next Section.

## 3. Applications

### 3.1 Colloidal actuation

Collective movement or self-assembly of colloidal particles has long been an interesting topic for the colloidal chemistry community but not until recently has attention been shifted to nano-/micro-swimmers and nanoactuators for the applications of drug delivery, nanosurgery and nanomachines.^75^ As colloidal swimmers are largely covered in previous reviews,^76^ here we mainly focus on static colloidal actuators that can change their shape or volume with external stimuli.^77^ Typical colloidal actuators mostly are composite materials that involve soft hydrogels and hard inorganics. Typically PNIPAM is applied for reversible (dis)assembly of Au NPs. In such a way, actuation can be realized in different dimensions, depending on their assembly configurations (Table 1). As most of the hydrogels have to work in aqueous environment, these thermosensitive nanoactuators are mainly applied as valves for microfluidics^78,79^ and actuators for DNA origami.^46^

**Table 1:** Au/PNIPAM colloidal actuation system of different types

Actuation type	1D		2D	3D
	Horizontal	Vertical		
Configuration	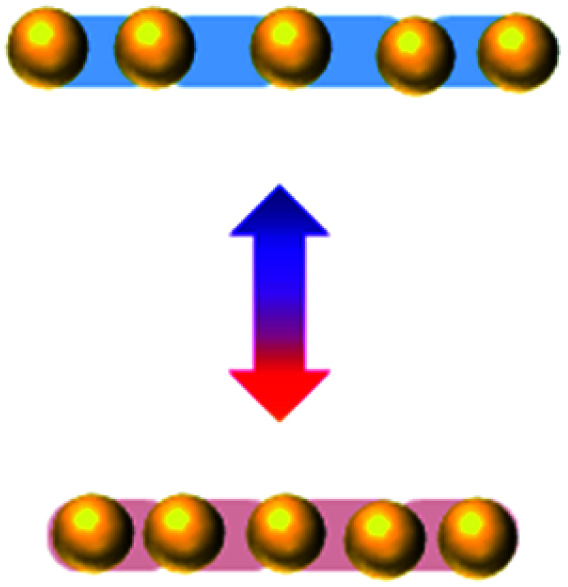	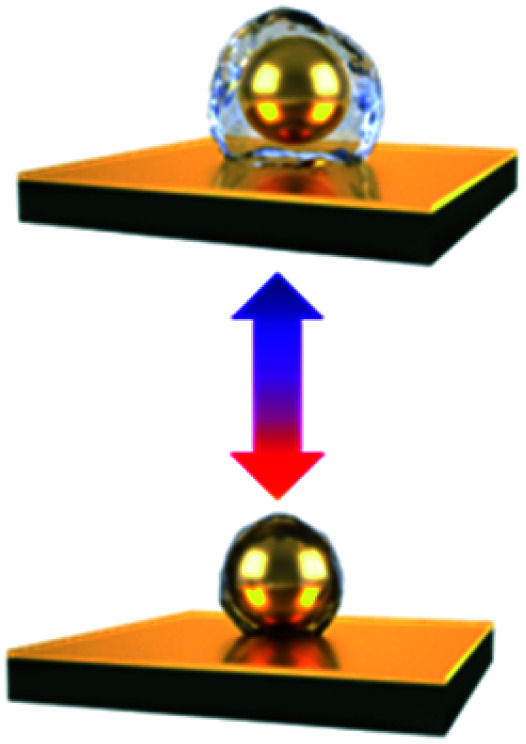	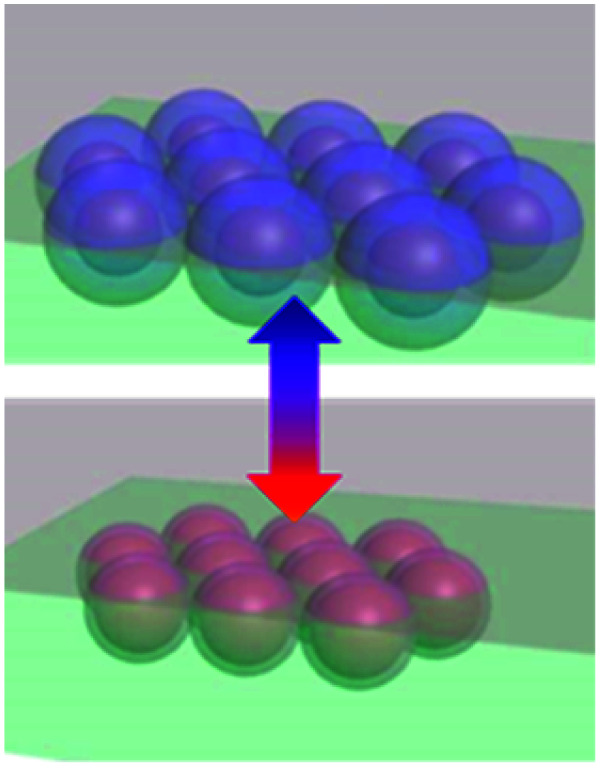	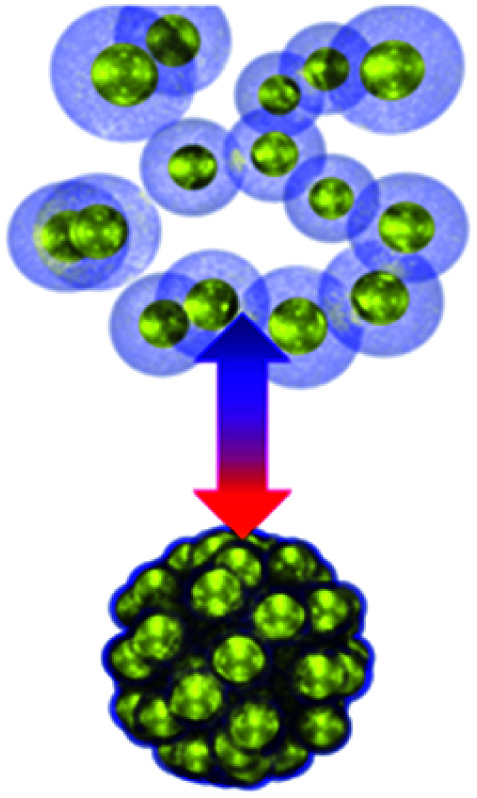
References	61	44 and 72	71	45, 60, 83 and 86

**Fig. 3:**
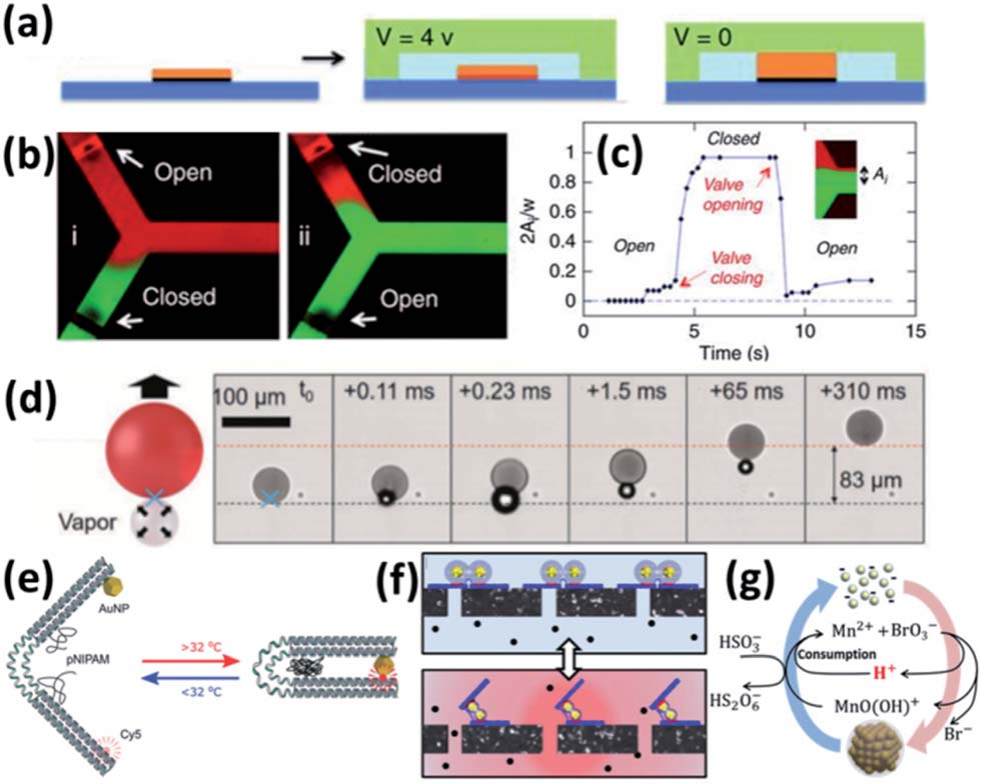
Colloidal actuation systems based on Au/PNIPAM. (a) Scheme of electric thermal valve action. (b) Fluorescence imaging of the microfluidics with electric thermo-controlled opening and closing of the valve. (c) Time response of the microvalve with electric thermal heating (modified from ref. 81. Copyright permission from Springer Nature). (d) Scheme of gas expansion mechanism for locomotion, with time-stamped microscope video frames taken with a high-speed camera (modified from ref. 82. Copyright permission from Wiley-VCH). (e) PNIPAM assisted actuation of DNA flexor (modified from ref. 46. Copyright permission from Wiley-VCH). (f) Scheme of Au/PNIPAM assisted actuation of filter valve (modified from ref. 45. Copyright permission from PNAS). (g) Chemomechanic oscillation with pH oscillation chemistry (modified from ref. 83. Copyright permission from ACS).

Early attempts by using photothermal effects to control microfluidic valves were based on composites of PNIPAM and photo-absorbing materials such as graphene^79^ and Au NPs,^69^ but the photothermal response is slow due to their large sizes. One way to improve its response speed is to use electrical thermal actuation.^80^ Here first patterned microelectrodes are made with PNIPAM brushes in the microfluidic channels. The thermal effect is induced by applying voltage to the electrodes, which then causes the shrinkage of PNIPAM (Fig. 3a). This works well for valve switching (Fig. 3b) and its speed can potentially be improved to ∼ms (Fig. 3c).^81^ But it requires the construction of wiring in the aqueous environment to form circuits (Fig. 3a), which increases the complexity of microfluidic chips. Another way to improve the photothermal response is to optically address the microfluidic valve with assistance of nanoplasmonics which provides unique advantages of simplicity, low cost, high integration and high speed. These are commonly made of Au/PNIPAM hybrid NPs which form clusters upon heating and expand upon cooling. This is a fast (∼μs) switching mechanism that can be remotely triggered by light, making it a light-induced actuating nanotransducer (ANT).^45^

These ANT NPs can be dispersed in microdroplets, which drive their directional movement due to the formation of Janus-type droplets.^82^ Such Janus droplets can accumulate heat on one side and generate microbubbles which propel their directional movement (Fig. 3d).

Another application of plasmonic nanoactuators is that they can help with DNA origami actuation, which is probed by changes of photoluminescence (Fig. 3e).^46^ The key is to engineer the DNA origami to accommodate dye molecules and thiol groups at the opposite ends and click chemistry for PNIPAM at the joint. This optical control of DNA origami flexors can potentially serve as optical gating for nanofiltering (Fig. 3f).^45^

Actuating Au NP/PNIPAM systems autonomously with constant energy input provides possibility for understanding and mimicking biological systems. With the coupling of pH oscillation reactions, Au@PNIPAM NPs show continuous cycling of aggregation and disaggregation (Fig. 3g) with energy efficiency up to 34%. Rather different from ANTs, the energy input here that continuously powers such actuation is supported by the chemical potentials, making it a chemomechanical energy transducer (CoMET).^83^

### 3.2 Tuneable plasmonics and metafilms

**Fig. 4:**
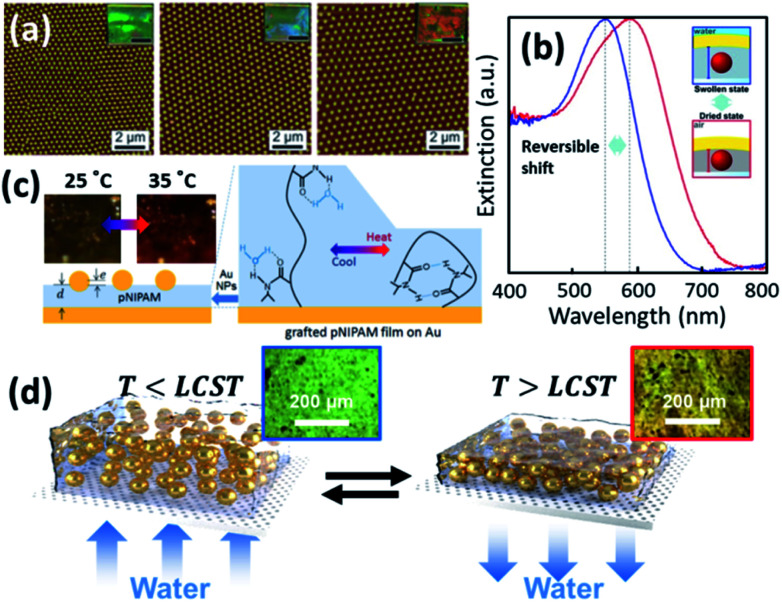
Thermosensitive plasmonics and metafilms based on Au/PNIPAM systems. (a) 2D arrays of Au/PNIPAM metafilms with tunable separation (modified from ref. 70. Copyright permission from RSC). (b) Reversible tuning of plasmon coupling with Au films overcoated on AuNP/PNIPAM hybrid films (modified from ref. 72. Copyright permission from ACS). (c) Change of NP optical interference with PNIPAM film thickness modulation (modified from ref. 49. Copyright permission from Wiley-VCH). (d) Switching of the reflection of hybrid Au/PNIPAM metafilms with temperature (modified from ref. 86. Copyright permission from Wiley-VCH).

As the plasmonic property of Au NPs is highly dependent on their separation and surrounding media,^9^ the colloidal actuation of plasmonic nanoparticles simultaneously induces changes of their optical properties. With colloidal actuation of PNIPAM, this has been largely applied for tuning of surface plasmons and metamaterials.^9^ The basic principle for the thermo-responsive tuning is the expansion and contraction of PNIPAM, which changes the separation between each Au NPs so the plasmon resonances changes accordingly. Another tuning strategy is keep the PNIPAM stable at fixed temperature but grow the Au cores within the PNIPAM so that their lattice constant is modified, which tunes the plasmon resonances (Fig. 4a).^70,84^ A further set of plasmonic nanoresonators such as Au NP on Au films,^85^ can also be tuned with PNIPAM actuation. The separation between Au NP and Au film changes in the out-of-plane direction with the expansion and contraction of the PNIPAM spacer, which reversibly tunes plasmon resonances (Fig. 4b).^72^ This plasmon tuning based on single Au NPs on mirror (NPoM) can be controlled with light due to the photothermal effect, which shows fast response (∼ms) due to rapid heating and cooling in a nanoscale volume.^44^

PNIPAM can also be applied for the tuning of plasmonic metafilms. A similar structure to Au NPoMs but with much thicker PNIPAM spacing layer (grown *via* atomic transfer radical polymerization) shows interference between Au NP scattering and Au film reflection. Such interference leads to different colours of the metafilms at different temperatures as the optical path difference changes with PNIPAM thickness (Fig. 4c).^49^ Plasmonic metafilms made of PNIPAM and Au NP composites also show dramatic color changes across the LCST of PNIPAM (Fig. 4d). These plasmonic metal films again can be tuned with an unfocussed halogen lamp as it produces strong photothermal heating within the films, thus contracting the composite metafilms. As a result, the filling ratio of the metafilms increases which results in a redshift of plasmons.^86^

### 3.3 Bioimaging and sensing

As the change of plasmons also indicates the change of local environment, it can serve as a plasmonic ruler for sensing applications.^87,88^ The most direct sensing parameter is the temperature due to the thermosensitive polymer (PNIPAM). However, this is only applicable for qualitative sensing as it show a nonlinear plasmon response *vs.* temperature with strong hysteresis.^60^ This can be improved for quantitative temperature sensing if Au nanorods or bipyramids are used.^89^ Besides temperature sensing, other aspects that can potentially be exploited are the sensing of solvents, pH and ion concentration since these parameters influence the LCST of PNIPAM, which then affect plasmon shifts.

**Fig. 5:**
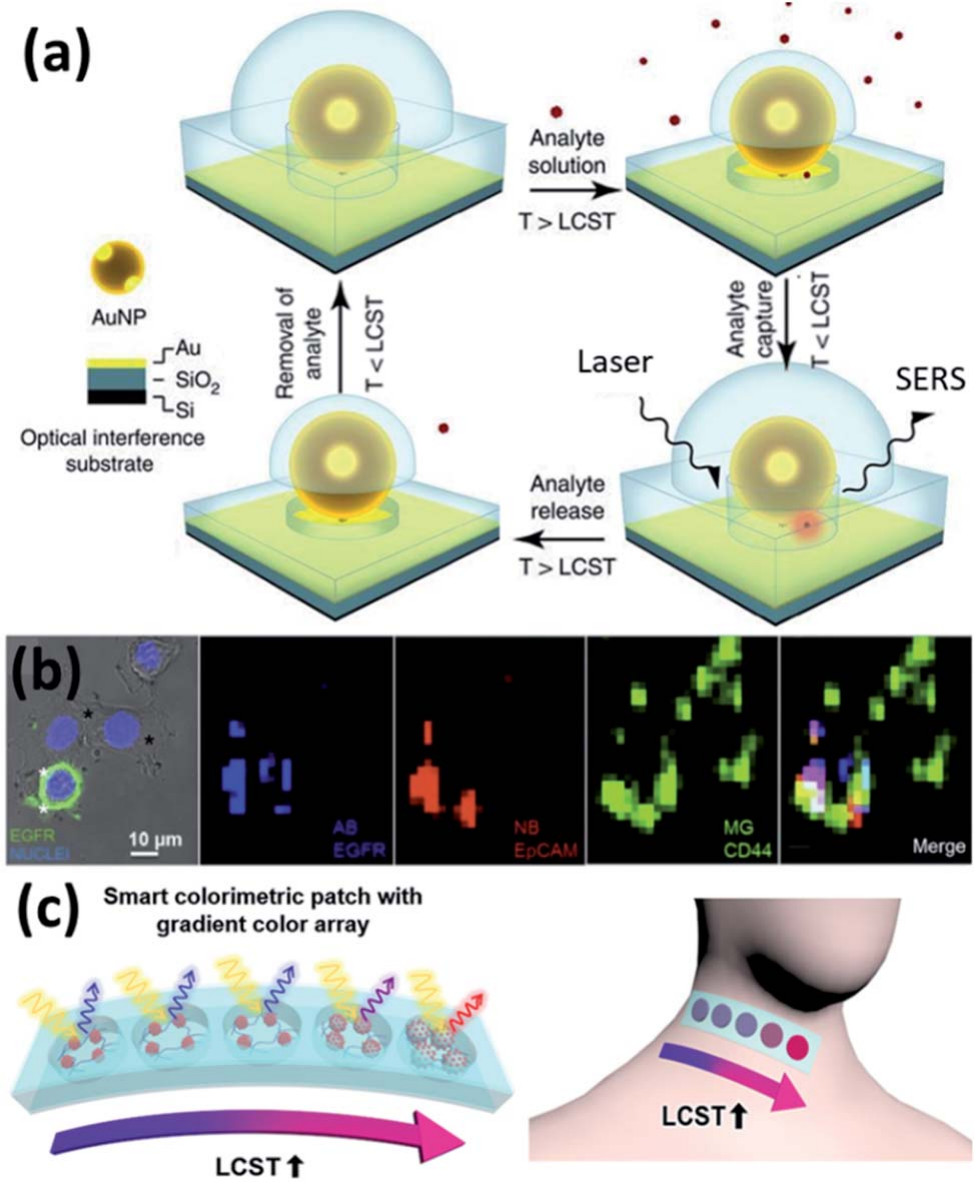
Plasmonic sensors based on Au/PNIPAM hybrids. (a) Au/PNIPAM nanostructure assisted single molecule detection (modified from ref. 26. Copyright permission from Springer Nature). (b) Au/PNIPAM hydrogel based SERS sensing and imaging of three tumor-associated biomarkers: epidermal growth factor receptor (EGFR), epithelial cell adhesion molecule (EpCAM), and homing cell adhesion molecule (CD44) (modified from ref. 74. Copyright permission from Wiley-VCH). (c) Au/PNIPAM hybrid gel films function as body temperature sensor (modified from ref. 48. Copyright permission from Springer Nature).

Another commonly applied sensing strategy with plasmons is to utilize their field enhancement effects to enable surface enhanced spectroscopies, such as Raman (SERS).^90^ The added value of using PNIPAM is that the temperature responsive shrinking and expansion of PNIPAM gels also provides a molecular trapping and release mechanism for SERS switching, which shows improved sensitivity (Fig. 5a).^26,47^ Such sensing can be applied in biological contexts enabling tags for cell imaging and cancer detection (Fig. 5b).^74^

Compared to SERS sensing, plasmonic colorimetric sensing provides an alternative route towards the sensing of chemicals that does not require high accuracy.^91^ It is much simpler and straightforward for on-site testing, which greatly improves the efficiency and convenience without any sophisticated instrumentation. Ko and colleagues developed a wearable colorimetric sensor for body temperature based on Au/PNIPAM hybrid gel films (Fig. 5c). They show temperature visualization over a wide temperature range of 25–40 °C with a response time of ∼1 s.^48^

## 4. Summary and outlook

We have reviewed developments of thermo-responsive plasmonic systems in recent years. Clearly this system is still developing with ever more interdisciplinary focus. Future efforts will likely focus more on the application side of this system. Actuators based on PNIPAM systems will be one of the main areas to optimise. These Au/PNIPAM hybrid systems are biocompatible and water soluble, and work over the body temperature range, making them ideal candidates for many bio-related applications. Current systems are either too slow or the forces too weak due to small Young's modulus of PNIPAM. One way to improve this is to incorporate activated nanogels into the system,^92^ while another way is to adopt metallic nanoparticles to boost the force output.^45^ The challenge is how to apply these in specific nanomechanical and biomechanical systems, which needs further nano-engineering. Colorimetric sensing based on plasmons is another intriguing area due to its sensitivity and simplicity. However, the colour saturation of plasmons is poor due to the high losses of metals, which potentially can be made up by combining particles with gain materials. Sensing should not be restricted to simple temperature ranges as it can potentially also involve many other physicochemical parameters such as solvent, ion strength, pH and refractive index. As for plasmon tuning, this Au/PNIPAM system seems to be not immediately attractive as they are less compatible with electronics due to their working environment (with water). Thus applications in microfluidics, biomedicine and drug delivery are a sensible direction since both Au and PNIPAM are biocompatible and functional in the aqueous phase. This smart plasmonic system will continue to grow with many new applications such as microfluidics, SERS tags, nanomedicine and nanomachines.^93^

## Conflicts of interest

There are no conflicts to declare.

## Supplementary Material
